# Men’s attitudes on gender equality and their contraceptive use in Uttar Pradesh India

**DOI:** 10.1186/1742-4755-11-41

**Published:** 2014-06-04

**Authors:** Anurag Mishra, Priya Nanda, Ilene S Speizer, Lisa M Calhoun, Allison Zimmerman, Rochak Bhardwaj

**Affiliations:** 1 International Center for Research on Women (ICRW), New Delhi, India; 2 Carolina Population Center, University of North Carolina at Chapel Hill, Chapel Hill, NC, USA; 3 Department of Maternal and Child Health, Gillings School of Global Public Health, University of North Carolina at Chapel Hill, Chapel Hill, NC, USA; 4 Ex-Consultant and Research Fellow with ICRW, New Delhi, India; 5 Urban Health Initiative (UHI), New Delhi, FHI360, India

## Abstract

**Background:**

Men play crucial role in contraceptive decision-making, particularly in highly gender-stratified populations. Past research examined men’s attitudes toward fertility and contraception and the association with actual contraceptive practices. More research is needed on whether men’s attitudes on gender equality are associated with contraceptive behaviors; this is the objective of this study.

**Methods:**

This study uses baseline data of the Measurement, Learning, and Evaluation (MLE) Project for the Urban Health Initiative in Uttar Pradesh, India. Data were collected from a representative sample of 6,431 currently married men in four cities of the state. Outcomes are current use of contraception and contraceptive method choice. Key independent variables are three gender measures: men’s attitudes toward gender equality, gender sensitive decision making, and restrictions on wife’s mobility. Multivariate analyses are used to identify the association between the gender measures and contraceptive use.

**Results:**

Most men have high or moderate levels of gender sensitive decision-making, have low to moderate levels of restrictions on wife’s mobility, and have moderate to high levels of gender equitable attitudes in all four cities. Gender sensitive decision making and equitable attitudes show significant positive association and restrictions on wife’s mobility showed significant negative relationship with current contraceptive use.

**Conclusion:**

The study demonstrates that contraceptive programs need to engage men and address gender equitable attitudes; this can be done through peer outreach (interpersonal communication) or via mass media. Engaging men to be more gender equal may have an influence beyond contraceptive use in contexts where men play a crucial role in household decision-making.

## 

The role of gender equality, when women and men have equal participation in decision-making and control of resources, and equal value and treatment, has been shown to influence a number of maternal and child health outcomes positively [1-9]. Most of the research that has focused on decision making around contraception has focused on how women’s lower autonomy in these decisions, and men’s attitudes on family planning have a strong influence on contraceptive use. Particularly, studies from South Asia have shown that women’s self-reported decision making (autonomy) has a significant effect on women’s uptake of antenatal care and tetanus toxoid immunization, and other childhood immunizations [2,4,7,10], as well as contraceptive use [11-17]. Research from Sub-Saharan Africa also shows a relationship such that women’s fertility preferences and contraceptive adoption are influenced by husband’s influence on women’s decision-making as well as their own attitudes [16,18-25].

The majority of studies demonstrating an influence of husband’s attitudes and preferences on women’s behaviors have focused on women’s perceptions of men’s attitudes toward fertility and contraception, and demonstrate the lack of discussion between husbands and wives on contraception [13,19,26-29]. Much of this research has lacked data directly from men on their own attitudes. Focusing on women’s data without attention to men’s data may result in a lack of crucial information. On the other side, the few studies that examine the relationship between men’s own attitudes and their contraceptive use, have focused on the role of men’s awareness, knowledge, perceptions of family planning on reasons for non-use of contraception [30,31]. There is limited research that explores the relationship between men’s attitudes and behavior around gender equality, i.e. men’s attitudes on whether and what kinds of decision making freedom women should have, and contraceptive outcomes. Interestingly, there are studies that have shown that men’s attitudes about gender equality are associated with condom use to prevent HIV [32-36].

Another dimension of contraceptive use research indicates a disparity between men’s and women’s use where men tend to report higher contraceptive use than women [27,37-41]. These studies underscore that higher reporting by men may be due to their contraceptive use outside marriage, and that men tend to give more socially acceptable responses than women [42,43]. Besides the socially appropriate responses, the men and women may also perceive decision making autonomy differently due to the real gender differentials in power and relationships, particularly in patriarchal societies [2,25]. One study from rural Uttar Pradesh, India examining the concordance between men’s and women’s reports about autonomy using indices of mobility, decision-making and access to economic resources demonstrated that husbands perceived their wives’ autonomy to be greater than the wives perceived it to be. The study found that husbands’ perceptions of autonomy were actually a better predictor of outcomes on reproductive behavior than wives’ perceptions of their own autonomy [27], underscoring the need to explore men’s attitudes toward gender equality and contraceptive use more closely.

Investigation of gender equality and contraceptive use among men in urban settings is important given the rapid growth of urban areas globally. Yet, the existing research provides limited insight into the relationship between men’s attitudes toward gender equality and contraception in the urban context, where sociocultural norms – including gender equality – and health care practices can vary significantly from the rural context and significant disparities are observed between the urban poor and urban rich [29,44]. We test the relationship between gender equality and contraceptive use in urban Uttar Pradesh; India’s most populous state with a population of over 200 million people.

In this study, we hypothesize that men’s attitudes towards gender equality, the control they place on their wives and attitudes towards their freedoms, have an effect on couple current contraceptive use and choice of method. The data presented here are novel as they were gathered from men and relate to men’s own reported attitudes toward gender equality and the association with contraceptive use. The study tested whether men with more gender equitable attitudes are more likely to use modern methods. While there are studies that have shown that men’s attitudes about gender equality are associated with condom use to prevent HIV [16,32,33,36,45], we explore if the relationship also holds true outside the HIV context and hypothesize that among users, more gender equitable men are more likely to use methods that require more male involvement (e.g. condoms). Gender equality in this study is operationalized in terms of three key indicators: gender sensitive decision-making, restrictions on wife’s mobility and gender equitable attitudes. More specifically the study examines the following hypotheses –

•Urban men’s reported contraceptive use will be higher among men showing a greater level of gender sensitive decision making.

•Urban men’s reported contraceptive use will be higher when they report fewer restrictions on the wife’s mobility.

•Urban men’s reported contraceptive use is higher where they have higher levels of gender equitable attitudes.

•Use of methods that require male involvement, such as condoms, will be higher among men with higher gender equitable scores.

## Methodology

### Data

The Urban Reproductive Health Initiative (URHI) which is referred to as Urban Health Initiative (UHI) in Uttar Pradesh, India, is a multi-country program, including Nigeria, Kenya and Senegal – targeting the urban poor with the objective to improve contraceptive choice and increase access to high quality, voluntary contraception. The Measurement, Learning, and Evaluation (MLE) Project, led by the Carolina Population Center at the University of North Carolina in Chapel Hill, in association with the International Center for Research on Women (ICRW) was funded to undertake a rigorous impact evaluation of the UHI programs in Uttar Pradesh.

Baseline data from women were collected in 2010 in six cities of Uttar Pradesh using individual-level surveys and facility-based surveys in each of the six cities. The MLE Project and the study methods have been described previously [29]. This analysis utilizes baseline data from men, which was collected in four of the six study cities (Agra, Aligarh, Allahabad and Gorakhpur). Briefly, a two-stage sampling approach was used to collect a representative sample of men from each city. Cities were divided into slum and non-slum primary sampling units (PSU) based on satellite imagery and ground truthing, and a random sample of 64 slum PSU and 64 non-slum PSU were selected from each city. Listing and mapping exercises were carried out in each PSU to ascertain household eligibility, and a random sample of 20 eligible households was selected in each PSU based on a random number generator. All currently married men aged 18–54 in selected households were eligible for participation. Upon consenting for study participation, a structured questionnaire was administered to men by a trained male interviewer. Men were asked questions about their fertility desires, awareness of contraceptive methods, contraceptive use by themselves or their wife, and attitudes toward reproductive health decision-making. The men also answered questions about their attitudes and practices regarding gender equality within their marriage and in general.

A total of 6,431 currently married men aged 18–54 had complete interviews in the four study cities. This includes 1,281 men from Allahabad, 1,683 men from Agra, 1,873 men from Aligarh, and 1,594 men from Gorakhpur. The overall survey response rate was 88 percent.

### Outcome variables – contraceptive use

The outcomes of interest are current use of contraception and modern contraceptive method choice. These outcome variables were chosen in order to investigate whether men (or their wives) are using any form of contraception and, among users of modern methods, whether they are using a permanent method, condoms, or another modern method. We hypothesize that more equal attitudes will be related to use of any method as well as use of male centered methods, such as condom and Non-scalpel Vasectomy. The current use of contraceptives variable consists of three categories: non-users, traditional method users and modern method users. The coding was determined by two questions. First, the respondents were asked if they or their wives were currently doing something to avoid or delay getting pregnant. Those who responded “no” were coded as non-users. Those who responded “yes” were then asked what type(s) of method(s) they or their wife were currently using. Those using natural methods (including rhythm, periodic abstinence and withdrawal) were coded as traditional method users and those using surgical, hormonal or barrier methods (such as male and female condoms, IUDs, injections, the pill, male and female sterilization, spermicide, lactational amenorrhea (LAM), and emergency contraception) were coded as modern method users. Dual method users were categorized based on the most effective method they were using.

Responses to the two contraceptive use questions were also used to create the contraceptive method choice variable, which pertains only to users of modern methods. Modern method users were separated into three categories: permanent method (male and female sterilization) users, condom users, and other modern method users (IUD, pills, injections, spermicide, emergency contraception, and LAM). Due to the small number, (NSV) is included in permanent methods.

### Independent variables – gender measures

Three scale indicators were developed from questions on husband-wife power relations and attitudes on gender equality. These indicators are – Gender Sensitive Decision Making (GSDM), Restrictions on Wife’s Mobility (RWM) and Gender Equitable Men Scale (GEMS). The GSDM includes three questions, RWM includes four and GEMS includes 12 questions.

Decision-making is defined as gender sensitive when a woman has a say in deciding about matters that affect herself and her family – for this study, we consider decision-making to be gender equitable when a man reports that a decision is made solely by the woman or in partnership between both spouses. Three questions were asked to determine who makes decisions on: women seeking health care for themselves, major household purchases and women visiting friends/wife’s family. The questions were asked on a three-point scale of ‘mainly man decides,’ ‘mainly wife decides’ or ‘both jointly decide.’ The response ‘mainly man decides’ was coded as ‘0’. Responses that a decision is made jointly or by the woman were coded as ‘1’. The summative score for all three questions were further categorized as: 0 to 1 as low, 2 as medium and 3 as high gender sensitive decision-making. The scale reliability analysis (Cronbach’s alpha) showed a value of 0.70. The measure was also examined as a continuous measure and the results were the same as the ones shown in the analysis.

The restrictions on wife’s mobility were assessed based on men’s responses to whether they prohibit their wife from: working outside the home, having visits from people, visiting her friends, or visiting her family. Responses were recorded as ‘yes’ and ‘no’. All “yes” responses (that is, the man restricts his wife’s mobility) were given ‘1’ and “no” responses (that is, the man does not restrict his wife’s mobility) as ‘0’. A summative score for the four questions was calculated and categorized as: 0–1 as low, 2 as medium and 3–4 as high restrictions. The Cronbach’s alpha for the indicators was 0.57. The measure was also examined as a continuous measure and the results were the same as the ones shown in the analysis.

Attitudes on gender equality were gauged using responses to twelve attitudinal statements about sexuality, use of contraceptives, gender based violence and sharing of family responsibilities [46,47] (see Appendix A for the list of statements). The 12 items were a mix of attitudes that were gender equal and non-equal. For each statement, the response options were ‘agree’, ‘partially agree’ and ‘do not agree’. All the statements are made unidirectional before assigning the scores to a gender discriminatory statement as ‘2’ for do not agree, ‘1’ for partially agree and ‘0’ for agree. The Cronbach’s alpha for the indicators was 0.65. The summative score for all 12 statements obtained the range of ‘2 – 24’, which is further categorized as ‘2-10’ as low gender equality, ‘11-17’ as medium gender equality and ‘18-24’ as high gender equality. This methodology of creating a GEM scale has been used and validated in previous research in India as well as other countries [46,47]. The measure was also examined as a continuous variable and the results were the same as the ones shown in the analysis.

### Control variables

Several socio-demographic factors that may affect contraceptive use are controlled for in the adjusted analyses. These variables are: age group (18–29, 30–39, 40–49, 50 or more); education (no education, 1–8 years, 9–12 years, 12+ years); caste (scheduled caste/scheduled tribe (SC/ST), other backward caste (OBC), general); religion (Hindu/other^a^ vs. Muslim), parity (no children, one child, 2–3 children, 4–5 children, 6+ children); city and resident of slum or non-slum area. Considering the fertility intentions as mediating factor between the gender attitudes and contraceptive use, we also included fertility desire for having another child in future as: want no more, Want another two years later and want soon within two years. A wealth index was created across the 4 cities based on 27 household assets and housing characteristics. These included the ownership of a toilet, piped water, electricity, type of floor, type of roof, and numerous durable goods. We undertook principal components analysis and developed a factor score for the first factor. Once a factor score is obtained, the household sample is divided into weighted quintiles (groups of 20% each) categorized as: poorest, poor, medium, rich, and richest [29].

### Analytical methods

After conducting univariate analyses on demographics, gender measures, contraceptive use and method choice measures, we conducted bivariate analysis on the relationships between the gender measures and the contraceptive use and method choice outcomes. Finally, we conducted multivariate analyses to examine which of the gender and demographic factors are associated with each of the possible outcome categories. All analyses (univariate, bivariate and multivariate) are weighted using the full-sample weights across the four cities and adjusted for clustering in the sample.

The first outcome, current contraceptive use, has three categories: non-users, traditional method users, and modern method users; therefore multinomial logistic regression methods were used. The first model (Model 1) presents the unadjusted association between each gender measure and current contraceptive use. The next model (Model 2) examines the association between each gender measure and current use status, controlling for the social and demographic control variables. Finally, Model 3 includes all of the gender measures and the social and demographic controls. In Models 2 and 3, social and demographic controls were included but are not presented in the tables shown.

The second outcome examines method choice among modern method users. The categories are: sterilization users (male and female), condom users, and other modern method users. We use multinomial logistic regression analyses for this three-category outcome variable. As done for the analyses of current use, we undertake multiple analyses to examine the role of the gender measures on men’s contraceptive choices, first through an unadjusted analysis of each gender measure and contraceptive method choice, followed by an analysis that controls for the social and demographic control variables. Finally, we run the model that includes all of the gender measures and the social and demographic controls. As before, in Models 2 and 3, social and demographic controls were included but are not presented in the tables shown.

## Results

Table 1 presents the demographic characteristics of the men in the four study cities included in this analysis. Overall a total of 6431 men were interviewed across the four study cities. One-third of the men in the samples were educated for 9–12 years with another 27 percent that had more than 12 years of education. Less than one-fifth of men interviewed (18%) were Muslims. Ninety percent of the men in the sample had at least one child and at least one fifth had four or more children. As expected about 20 percent of men were in each of the wealth quintile groups. Sixteen percent of sampled men live in slums. Each city contributed around one-fourth of the sample, ranging from 20 percent in Allahabad to 29 percent in Aligarh. Finally, presented in Table 1 is the percentage of men with future fertility intentions. The majority of men said they do not want any more children (71%) with another 18 percent that wants to delay for two or more years.

**Table 1:** Background characteristics of samples of men from four study cities, Uttar Pradesh, India, 2010

***Characteristics***	**Percent of male respondents**
	**(n = 6431)**
**Age**	
18-29 years	22.7
30-39 years	34.9
40-49 years	32.8
50 or more	9.6
**Education**	
No education	14.1
1-8 years	26.4
9-11 years	32.7
12 or more years	26.8
**Religion**	
Hindu/other	82.5
Muslim	17.5
**Caste**	
Schedule caste/tribe	21.7
Other backward class	37.5
Others (general)	40.8
**Number of living children**	
No child	10.2
One child	17.4
2-3 children	46.5
4-5 children	19.9
More than 5 children	6.0
**Wealth**	
Poorest	18.5
Poor	19.5
Middle	19.9
Rich	21.6
Richest	20.5
**Residence**	
Slum	16.3
Non-slum	83.7
**City**	
Agra	26.2
Aligarh	29.0
Allahabad	20.1
Gorakhpur	24.7
**Desire for more children**	
Want child soon (within 2 years)	11.0
Want to delay 2 or more years	18.2
Do not want any more child	70.8

### Gender measures

About half of the men (48%) show high levels of gender sensitive decision-making, which means that for all three of the decisions, they make the decisions either jointly or their wife makes the decisions alone (see Table 2). Similarly, about half of men have low levels of restrictions on their wife’s mobility, meaning that men infrequently restrict their wife from working outside the home, having visits from people, visiting friends, or visiting her family. Across all cities, while a majority of men (59%) report to have moderate gender equal attitude, a little more than one-third of men (35%) have high gender equal attitudes (Table 2).

**Table 2:** Gender measures scales of men from four study cities, Uttar Pradesh, India, 2010

**Gender measures**	**Percent of male respondents**
	**(n = 6431)**
**Gender Sensitive Decision Making (GSDM)**	
Less sensitive	19.6
Moderately sensitive	32.3
Highly sensitive	48.1
**Restrictions of wife’s mobility**	
Less restrictive	50.3
Moderately restrictive	32.5
Highly restrictive	17.2
**Gender equal attitude scale**	
Less gender equal attitude	6.6
Moderate gender equal attitude	58.7
High gender equal attitude	34.7

### Current contraceptive use

Table 3 shows the percentage of currently married men aged 18–54 who are currently using a modern contraceptive method, a traditional contraceptive method or are non-users of contraceptives. Additionally, the contraceptive method mix for modern methods is also presented. Three-fifths of men report currently using a modern method and 13 percent of men report traditional method use.

**Table 3:** Modern contraceptive use and method mix among men in four study cities, Uttar Pradesh, India, 2010

**Contraceptive method mix**	**Percent of male respondents**
	**(n = 6431)**
**Modern method users#**	**60.2**
Pills	4.8
Condoms	33.2
Female sterilization	19.7
Male sterilization	0.7
IUD	1.3
Injections	0.3
Other modern methods	0.2
**Traditional method users**	**12.5**
**Non-users**	**27.3**

# Some men reported using dual methods. They are categorized based on the most effective method they were using.

The contraceptive method choice among men across the four cities shows a dominance of condom use–33 percent report condom use – followed by female sterilization – 20 percent. The use of other modern methods including IUD and injections is very low. Although injections are not yet included in the Government of India’s contraceptive basket and are only available through the private sector, a small number of men reported use of injections by their wives. The low use of IUD is notable despite the fact that it has been widely available through both public and private sector service delivery points for over two decades [11].

### Relationship with the gender measures

Table 4 presents the bivariate association between the three gender measures and men’s report of current modern contraceptive use. A significantly higher proportion of men with high GSDM reported contraceptive use as compared to the men with low GSDM (p < 0.05). The association of RWM with men’s reports of current contraceptive use shows that men with higher restrictions are significantly more likely to be users (p < 0.01). The GEMS scale shows a significant positive association with current contraceptive use across all cities. Men’s current contraceptive use increases as their gender equal attitudes score increases. While the results on GSDM and GEMS are consistent with the hypothesized pattern, the results with mobility restrictions show contrary results, and need to be looked at with multivariate analysis.

**Table 4:** Current contraceptive use and gender measures among men in four study cities, Uttar Pradesh, India, 2010

**Gender measures**	**Percentage of men who are current modern contraceptive method users by gender measure**
	**(n = 6431)**
**Gender Sensitive Decision Making (GSDM)**	
Less sensitive	56.8
Moderately sensitive	60.4
Highly sensitive	61.0*
**Restrictions of wife’s mobility**	
Less restrictive	61.8
Moderately restrictive	57.1
Highly restrictive	61.4**
**Gender equal attitude scale**	
Less gender equal attitude	55.7
Moderate gender equal attitude	59.0
High gender equal attitude	63.4***

*p < 0.05, **p < 0.01, ***p < 0.001.

### Multivariate analyses

#### Gender measures and current contraceptive use

In this section we explore the association between gender measures and current modern or traditional method use controlling for other factors associated with contraceptive use. A three-tier approach is taken and presented in Table 5. Firstly, in Model 1 we looked at the unadjusted beta-coefficients for the association between contraceptive use and each gender measure; in Model 2 we included the social and demographic controls and presented the adjusted results for the gender measures of interest; Model 3 presents the analysis with all gender measures and the social and demographic controls.

**Table 5:** Multinomial logistic regressions of gender measures and contraceptive use among men in four study cities, Uttar Pradesh, India, 2010

**Variables in equation**	**Modern methods vs. non user *(Ref.)* β (SE)**	**Traditional methods vs. non-user *(Ref.)* β (SE)**	**Modern methods vs. traditional *(Ref.)* β (SE)**
**Gender sensitive decision making – Unadjusted – Model 1**			
**Gender sensitive decision making (GSDM)**			
Highly sensitive	0.09 (0.08)	−0.14 (0.12)	0.05 (0.11)
Moderately sensitive	0.04 (0.08)	0.18 (0.12)	−0.15 (0.11)
*Less sensitive (Ref.)*			
**Gender sensitive decision making - Adjusted for Demographics† − Model 2**			
**Gender sensitive decision making (GSDM)**			
Highly sensitive	0.18 (0.09)*	−0.23 (0.12)	0.06 (0.11)
Moderately sensitive	0.03 (0.09)	0.20 (0.13)	−0.17 (0.11)
*Less sensitive (Ref.)*			
**Restrictions on Wife’s Mobility – Unadjusted – Model 1**			
**Restrictions on wife’s mobility**			
Less restrictive	−0.08 (0.08)	−0.27 (0.12)*	0.19 (0.10)+
Moderately restrictive	−0.36 (0.09)***	−0.54 (0.13)***	0.18 (0.11)
*Highly restrictive (Ref.)*			
**Restrictions on Wife’s Mobility - Adjusted for Demographic† s – Model 2**			
**Restrictions on wife’s mobility**			
Less restrictive	−0.29 (0.10)**	−0.61 (0.13)***	0.32 (0.11)**
Moderately restrictive	−0.40 (0.10)***	−0.63 (0.13)***	0.23 (0.12)*
*Highly restrictive (Ref.)*			
**Gender Equal Attitude Scale – Unadjusted – Model 1**			
**Gender equal attitude scale**			
High gender equal attitude	0.45 (0.12)***	0.48 (0.19)*	−0.03 (0.18)
Moderate gender equal attitude	0.27 (0.11)*	0.49 (0.18)**	−0.22 (0.18)
*Less gender* equal *attitude (Ref.)*			
**Gender Equal Attitude Scale - Adjusted for Demographics† − Model 2**			
**Gender equal attitude scale**			
High gender equal attitude	0.21 (0.14)	0.04 (0.21)	0.18 (0.19)
Moderate gender equal attitude	0.24 (0.13)+	0.41 (0.19)*	−0.18 (0.18)
*Less gender equal attitude (Ref.)*			
**Gender Measures Adjusted for Demographics**† **and Other Gender Measures – Model 3**			
**Gender sensitive decision making (GSDM)**			
Highly sensitive	0.17 (0.10)	−0.15 (0.13)	−0.02 (0.11)
Moderately sensitive	0.05 (0.10)	0.22 (0.13)	−0.18 (0.12)
*Less sensitive (Ref.)*			
**Restrictions on wife’s mobility**			
Less restrictive	−0.32 (0.10)**	−0.58 (0.13)***	0.26 (0.11)*
Moderately restrictive	−0.44 (0.10)***	−0.68 (0.14)***	0.24 (0.12)*
*Highly restrictive (Ref.)*			
**Gender equal attitude scale**			
High gender equal attitude	0.24 (0.14)+	0.09 (0.21)	0.15 (0.19)
Moderate gender equal attitude	0.21 (0.13)+	0.37(0.20)+	−0.16 (0.18)
*Less gender equal attitude (Ref.)*			

†Demographics include Age, Education, Caste, Religion, Parity, Wealth, City and Residence (slum and non-slum) and Fertility intention; +p < 0.1, *p < 0.05, **p < 0.01, ***p < 0.001.

The multivariate analysis examining gender sensitive decision making shows a significant association between the high GSDM score and current modern contraceptive use as compared to non-users. There is no significant association between the GSDM and traditional contraceptive use as compared to non-users. While unadjusted coefficients are non-significant, the coefficients adjusted for social and demographic variables are significant for men with high GSDM score compared to the low GSDM score.

Interesting findings are observed in the analysis of the relationship between RWM score and their current modern and traditional contraceptive use. The general conjecture would be that less restrictive men are more likely to be contraceptive users. On the contrary, the adjusted coefficients for the restricted mobility measure shows that men with less restrictive mobility are significantly less likely to be modern or traditional method users than non-users. The adjusted coefficients for restricted mobility, for comparison between modern and traditional method users, show that men with less and moderate restrictive mobility, compared with men with high restrictive mobility, are significantly less likely be the modern method users than traditional method users.

The unadjusted coefficients for GEMS score show that, compared to the men with less gender equal attitudes, the men with medium or high gender equal attitudes are significantly more likely be current modern method users than non-users. Likewise, men with moderate or high gender equal attitudes are significantly more likely to be traditional method users than non-users as compared to those with less gender equal attitudes. No significant difference is found by gender equal attitudes level on whether a man is a modern or traditional method user. The relationship between the GEMS score and current modern or traditional method use is weak after controlling for the social and demographic factors. For both modern and traditional method users compared to non-users, only the moderate category remains significant.

Model 3, which is also shown in Table 5, presents the adjusted coefficients for all three of the gender measures controlling for the social and demographic factors. Broadly, the results are similar to those presented above, i.e. the gender measures remain in the same direction, although some of the significance is attenuated. Men with high or moderate gender equal attitudes are more likely to use modern methods as compared to non-users. Similarly, the men with less or moderately restrictive attitudes on mobility are less likely to use modern or traditional methods than non-users.

#### Socio-demographic characteristics and current contraceptive use

Current contraceptive use models controlling for socio-demographic characteristics show that the relationships are generally in the expected direction, such that men who are younger are more likely to use modern methods than be non-users or use traditional methods as compared to men who are older (data not shown). Less educated men are less likely to use modern methods and more likely to be a non-user than more educated men. Likewise, men with fewer children are less likely to use modern methods and more likely to be a non-user than men with six or more children. Similar to the results shown by Speizer et al. with female data [29], the poorest men are the least likely to use modern methods. The key city-specific difference was that men from Aligarh are more likely to use traditional methods than modern methods and more likely to be non-users than be traditional method users than men from Gorakhpur (the reference group). The differences are all significant at the p < 0.05 level and remain similar in models that include the gender measures (not shown).^b^ The association between men’s desire to have more children in future and modern method use is significant; the men with desire to have no more children or want to delay child bearing for two or more years are more likely be the current contraceptive users.

### Current modern method choice

In this section we present the multivariate analysis of current modern contraceptive method choice and the association with the three gender measures as well as with the social and demographic covariates.

#### Gender measures and contraceptive method choice

The unadjusted coefficients for gender sensitive decision making show that with reference to men with less GSDM score, the men with high or moderate GSDM score are significantly more likely to use condoms than sterilization (Table 6). Comparing the other modern method use against sterilization, the men with moderate GSDM score are significantly more likely to use other modern methods than sterilization (see Model 1). The condom use against other modern methods shows that with reference to the men with low GSDM, the men with high GSDM are significantly more likely to use condoms and men with moderate GSDM are significantly less likely to use condoms than other modern methods. When adjusted for social and demographic factors (see Model 2), the results remain generally similar to the unadjusted results.

**Table 6:** Multinomial logistic regressions of gender measures and current contraceptive method mix among men (modern method users) in four study cities, Uttar Pradesh, India, 2010

**Variables in equation**	**Condom vs. sterilization *(Ref.)* β (SE)**	**Other modern methods vs. sterilization *(Ref.) *** **β (SE)**	**Condom vs. other modern methods *(Ref.)* β (SE)**
**Gender sensitive decision making –Unadjusted – Model 1**			
**Gender sensitive decision making (GSDM)**			
Highly sensitive	0.44 (0.09)***	0.11 (0.16)	0.33 (0.15)*
Moderately sensitive	0.29 (0.10)**	0.68 (0.16)***	−0.39 (0.15)*
*Less sensitive (Ref.)*			
**Gender sensitive decision making - Adjusted for Demographics† − Model 2**			
**Gender sensitive decision making (GSDM)**			
Highly sensitive	0.36 (0.11)*	0.09 (0.17)	0.26 (0.16)+
Moderately sensitive	0.18 (0.11)	0.65 (0.17)***	−0.47 (0.16)**
*Less sensitive (Ref.)*			
**Restrictions on Wife’s Mobility–Unadjusted – Model 1**			
**Restrictions on wife’s mobility**			
Less restrictive	−0.55 (0.10)***	−0.78 (0.15)***	0.22 (0.14)
Moderately restrictive	−0.50 (0.11)***	−0.75 (0.16)***	0.25 (0.15)
*Highly restrictive (Ref.)*			
**Restrictions on Wife’s Mobility - Adjusted for Demographics† − Model 2**			
**Restrictions on wife’s mobility**			
Less restrictive	−0.43 (0.12)***	−0.60 (0.16)***	0.17 (0.14)
Moderately restrictive	−0.49 (0.13)**	−0.74 (0.18)***	0.26 (0.15)
*Highly restrictive (Ref.)*			
**Gender Equal Attitude Scale –Unadjusted – Model 1**			
**Gender equal attitude scale**			
High gender equal attitude	0.63 (0.15)***	0.51 (0.25)*	0.12 (0.24)
Moderate gender equal attitude	0.46 (0.15)**	0.34 (0.24)	0.13 (0.24)
*Less gender equal attitude (Ref.)*			
**Gender Equal Attitude Scale - Adjusted for Demographics† − Model 2**			
**Gender equal attitude scale**			
High gender equal attitude	0.08 (0.20)	0.24 (0.27)	−0.17 (0.26)
Moderate gender equal attitude	0.20 (0.16)	0.21 (0.26)	−0.01 (0.25)
*Less gender equal attitude (Ref.)*			
**All Gender Measures – Adjusted for Demographics† and other Gender Measures – Model 3**			
**Gender sensitive decision making (GSDM)**			
Highly sensitive	0.41 (0.11)***	0.10 (0.17)	0.31 (0.16)*
Moderately sensitive	0.20 (0.12)+	0.70 (0.17)***	−0.50 (0.16)**
*Less sensitive (Ref.)*			
**Restrictions on wife**			
Less restrictive	−0.42 (0.12)***	−0.54 (0.17)***	0.11 (0.15)
Moderately restrictive	−0.49 (0.13)***	−0.75 (0.18)***	0.26 (0.16)
*Highly restrictive (Ref.)*			
**GE gender equal attitude scale**			
High gender equal attitude	0.08 (0.18)	0.20 (0.27)	−0.12 (0.26)
Moderate gender equal attitude	0.26 (0.17)	0.10 (0.26)	−0.16 (0.25)
*Less gender equal attitude (Ref.)*			

†Model for adjusted coefficients controls for Age, Education, Caste, Religion, Parity and Wealth, City, Residence (slum/non-slum), Fertility intention and other gender equity measures – gender sensitive decision making and GEM scale; *p < 0.05, **p < 0.01, ***p < 0.001.

The analysis of RWM score and method choice are also presented in Table 6. The analysis shows a similar, unexpected pattern as was found for current contraceptive use. Both unadjusted and adjusted coefficients for restrictive mobility suggest that with reference to highly restrictive men, the men with less and moderate restrictions are significantly less likely to use condoms or other modern methods as compared to sterilization. Comparison of condom use against other modern methods did not show a significant association with restrictions on wife’s mobility (Table 6).

The unadjusted coefficients for GEMS score and its association with method choice show expected results such that men with higher gender equal attitudes are significantly more likely to use condoms or other modern methods rather than be sterilization users as compared to men with lower gender equal attitudes. However, when social and demographic controls are included, no significant association is observed between the GEMS score and men’s likelihood to use condoms or other modern methods over sterilization.

Finally, at the bottom of Table 6, the results including all of the gender measures controlling for the social and demographic variables are presented. The results of these analyses are similar to those above indicating that GSDM is positively and RWM is negatively associated with method choice, particularly with choice of condoms or other modern methods over sterilization. The results of the other demographic variables remain similar with the addition of the gender measures (not shown).

#### Socio-demographic characteristics and current contraceptive method choice

The multinomial results for contraceptive choice among current modern contraceptive users show the results as expected; younger men and men with fewer children are more likely to be condom users or other modern method users as compared to sterilization users. Less educated men are less likely to be condom users or other modern method users as compared to sterilization users. Poorer men and men from slums are more likely to have sterilized wives than to be condom or other modern method users. The men who want no more children or those who want to delay having another child for two years or more are more likely to use other modern contraceptive methods then those wanted the next child within two years period (not shown).

## Discussion

The study found that men’s attitudes on gender equality play a significant role in use of contraception in urban settings. The results are not consistent across the three gender measures, and not always in the hypothesized direction. There exists a positive and significant relationship between men’s gender sensitive decision making and gender equal attitudes scores with their likelihood of using a modern contraceptive method, but restrictions on mobility is not positively associated with contraceptive use.

The univariate results of the three measures of gender equality suggest that most men have high or moderate levels of gender sensitive decision-making, have low to moderate levels of restrictions on wife’s mobility, and have moderate to high levels of gender equitable attitudes. Men’s reported modern method use is around 60 percent, with a dominance of condom use followed by female sterilization. The results of cross tabulations of gender equality with contraceptive use are not consistent across the three measures. The gender sensitive decision making scale shows a significant and positive association with current contraceptive use. Furthermore, gender equal attitudes also showed a positive and significant relationship, and restrictions on wife’s mobility showed a negative and significant relationship with current contraceptive use. These relationships sustain in the multivariate analysis adjusting for demographic variables. For the analyses of contraceptive method mix, we demonstrate that men with higher gender sensitive decision making are more likely to be condom users as compared to sterilization users. Additionally men with less restrictive attitudes on wife’s mobility are less likely to be condom or other modern method users and thus more likely to be using sterilization.

The multivariate results are also not consistent across all measures. Similar to studies using women’s data that have found a significant relationship between women’s decision-making autonomy and current contraceptive use, the relationship between gender sensitive decision-making and current contraceptive use as reported by men is also significant [12,15,48,49]. Jejeebhoy [27] showed that in rural UP, a setting where women are often not empowered to make fertility decisions on their own, men’s perceptions of their wives autonomy were more influential on their reproductive behavior than women’s perceptions of their own autonomy. This underscores the importance of further exploring men’s attitudes and perceptions of autonomy and contraceptive use in varying contexts in UP. Of interest in the Jejeebhoy paper is that their results also do not always go in the expected directions for each of the autonomy measures studied, and none of the variables exploring husband’s perceptions of women’s autonomy are significant in the contraceptive use model. Decision-making authority and mobility and current contraceptive use echo the results of this analysis. While our study supports the overall findings of some of the other cited studies using women’s data that have found a significant relationship between women’s decision-making autonomy and current contraceptive use [12,15,27,49,50], its significant relationship is limited to only modern method use.

Interestingly, the relationship between restrictions on wife’s mobility and current contraceptive use in this study is in the opposite direction to what was hypothesized. While some studies among women have found no significant relationship between mobility and contraceptive use, others have shown the relationship to be positive [43,51,52]. In our study, higher restrictions on wife’s mobility were associated with *higher* contraceptive use and more use of condoms and other modern methods. This may strengthen prior assertions that though lower mobility has a significant relationship with inhibiting inter-spousal communication regarding contraceptive use [27], it does not seem to be associated with actual behaviors around contraceptive use [49]. Mobility is influenced by the context in which a man or a woman lives. For example, women may face restrictions on their mobility due to the lack of safety in their external environment, at the same time women stepping out may not be culturally acceptable. One study on gatekeepers’ attitude on contraceptive use in rural Uttarakhand (part of Uttar Pradesh earlier) indicated that while they were rigid on women’s mobility for work, they were supportive of women using modern contraceptives [53]. However, the mobility in urban context may be different than rural areas where things are closer possibly, and while women may experience lower mobility, they may have access to services through components that are not culturally rigid, such as child health visits. This may be why mobility produces contrary results to gender sensitive decision making that had a positive and significant effect on modern method use versus non-use in the adjusted models.

Like other studies, our study also shows a positive and significant association between parity and intention to limit childbearing in future and current contraceptive use [17,54,55]. We take these fertility variables into account when examining the relationship between the gender measures and current contraceptive use by controlling for them in adjusted models.

This study has a number of strengths and limitations. In terms of its strengths, first, the study deals with the contraceptive use and choices of urban men. Much of the existing body of research on men in the contraceptive domain is limited to demographic and economic factors associated with men’s reported contraceptive use. The knowledge of men’s gender attitudes and whether these are associated with contraceptive use, especially for the socio-cultural contexts where male dominance is prevalent, is very limited. The focus on urban men in this study gives valuable insights about contraceptive use and its determinants and it will help inform the policies and programs targeting increased contraceptive use in urban settings.

This study is also novel because it examines gender equal measures of men’s attitudes and behaviors (mobility and decision-making men’s attitudes on gender equality). Typically men’s attitudes have been measured as proxy measures reported by women (or wives); this is likely to be biased data because men’s attitudes reported by women are influenced by women’s own perceptions. Therefore the addition of men’s own attitudes, particularly on gender equality, provides unique insights into fertility behavior from the male perspective.

One of the key limitations of this study is that men may over report contraceptive use in settings where there are increased numbers of programs on contraception [56]. Comparing reported contraceptive use of women [29] and men in the same urban sites demonstrates that use levels are reported as higher among men; this may be indicative of over-reporting among men or under-reporting among women. Men were asked about contraceptive use by themselves or their partner; thus some men may have reported contraceptive use with an outside partner, thus inflating the overall contraceptive use prevalence (particularly condoms). Another limitation of this analysis is how the mobility measure is constructed. Although, the three questions used in this study to measure the domain of mobility are widely used in other studies, mobility is often context specific and sensitive to social-environmental factors. Next, the data may be limited to the extent that the MLE survey, like other large scale surveys such as the India National Family Health Survey (NFHS), uses structured interviews that do not probe deeply into gender equality topics and do not include open ended questions to get greater insights [29,57]. Qualitative data would have added critical insights and could have been used to supplement this study to provide answers to the questions of why and how certain measures of gender equality are associated with contraceptive use. Lastly, this paper includes only men’s reports and thus could be criticized for not providing women’s perspectives. While female data were collected in the MLE study, it was from different households so it was not possible to create a couple-sample for this analysis. Another limitation of the study is that we have data on men’s reports of decision-making and mobility which may be limited in terms of representing the overall household decision making, which is often used in the gender research using women’s data [27]. Overall the study endorses the importance of understanding men’s gender attitudes to help understand men’s contraceptive behaviors. Further studies should expand this work and examine the couple-level gender equality measures on couple-level contraceptive use.

This study demonstrates that promoting the adoption of gender equal attitudes among men can achieve positive results such as increasing modern contraceptive use among men (and women). Since the relations of gender sensitive decision making and gender equal attitudes with contraceptive use are positive and significant, the study suggests that contraceptive programs should engage with men to enhance their attitudes towards gender equality. This can be done through peer outreach (interpersonal communication) or via the mass media. Such interventions may have an influence beyond contraceptive use to improve health and social outcomes for women such as reducing gender based violence, preventing HIV infection and enhancing women’s participation in economic activities and thus their overall empowerment. The study thus frames an agenda for future research on men, particularly to understand how much men are changing toward accepting and practicing gender equality and how policies and programs can speed up that change.

## Endnotes

^a^Note that less than 1% was non Hindu (Budhhist, Christian, Jain, etc.); these men were included in this category.

^b^Contact first author for full models.

## Appendix –A: statements showing men’s attitude on gender issues (for gender equal attitude scale)

1. You don’t talk about sex, you just do it.

2. Women who carry condoms on them are “easy”.

3. Changing diapers, giving the kids a bath, and feeding the kids are the mother’s responsibility.

4. It is a woman’s responsibility to avoid getting pregnant.

5. A man should have the final word about decisions in his home.

6. A woman should tolerate violence in order to keep her family together.

7. I would be outraged if my wife asked me to use a condom.

8. A couple should decide together if they want to have children.

9. In my opinion, a woman can suggest using condoms just like a man can.

10. If a man gets a woman pregnant, the child is the responsibility of both.

11. It is important that a father is present in the lives of his children, even if he is no longer with the mother.

12. A man and a woman should decide together what type of contraceptive to use.

## Competing interests

The authors declare that they have no competing interests.

## Authors’ contributions

AM conceptualized this research article, carried out the analysis and wrote the analysis. PN, IS and LC contributed to putting in the initial section and discussions. AZ and RB contributed in putting together the graphs and tables and review of manuscript drafts. All authors read and approved the final manuscript.

## References

[B1] Acharya DR Bell JS Simkhada P van Teijlingen ER Regmi PR Women’s autonomy in household decision-making: a demographic study in Nepal Reprod Health 2010 7 15 doi:10.1186/1742-4755-7-15 20630107 10.1186/1742-4755-7-15 PMC2914657

[B2] Allendorf K Couples' reports of women's autonomy and health‒care use in Nepal Stud Fam Plann 2007 38 1 35 46 10.1111/j.1728-4465.2007.00114.x 17385381

[B3] Blanc AK The effect of power in sexual relationship on sexual and reproductive health: An examination of the evidence Stud Fam Plann 2001 32 3 189 213 10.1111/j.1728-4465.2001.00189.x 11677692

[B4] Bloom SS Gupta MD Wypij D Dimensions of women's autonomy and the influence on maternal health care utilization in a North Indian city Demography 2001 38 1 67 78 10.1353/dem.2001.0001 11227846

[B5] Canadian International Development Agency (CIDA) Gender Equality Policy and Tools: Cida’s Policy on Gender Equality 2010 Canada http://www.cida.gc.ca

[B6] Casterline BJ Sathar ZA Haque-ul M Obstacles to contraceptive use in Pakistan: a study in Punjab Stud Fam Plann 2001 32 2 95 110 10.1111/j.1728-4465.2001.00095.x 11449867

[B7] Furuta M Salway S Women's position within the household as a determinant of maternal health care use in Nepal Int Fam Plan Perspect 2006 32 1 17 27 10.1363/3201706 16723298

[B8] Hausmann R Tyson LD Zahidi S The Global Gender Gap Report 2012 Geneva: World Economic Forum

[B9] Pulerwitz J Barker J Measuring attitudes toward gender norms among young men in Brazil Men and Masculinities 2008 10 3 322 338

[B10] Gupta MD Life course perspectives on women's autonomy and health outcomes American Anthropologist 1995 97 3 481 491 10.1525/aa.1995.97.3.02a00070

[B11] Khan ME Kar SS Desai VB Patel P Itare BP Garge S Increasing the Accessibility, Acceptability and Use of the IUD in Gujarat, India Frontiers Final Report 2008 New Delhi, India: Population Council

[B12] Khan ME Patel B Male Involvement in Family Planning: A Knowledge Attitude Behavior and Practice Survey of Agra District 1997 New Delhi: Population Council

[B13] Mason KO Smith HL Husband’s versus wives’s fertility goals and use of contraception: the influence of gender context in five Asian countries Demography 2000 37 3 299 311 10.2307/2648043 10953805

[B14] Morgan SP Niraula BB Gender inequality and fertility in two Nepali villages Popul Dev Rev 1995 21 3 541 561 10.2307/2137749

[B15] Moursund A Kravdal O Individual and community effects of women's education and autonomy on contraceptive use in India Popul Stud 2003 57 3 285 301 10.1080/0032472032000137817 22010867

[B16] Niraula BB Lawoti D Women's autonomy and reproductive behavior in two urban areas of Nepal Contributions to Nepalese Studies, Special Issue on Fertility Transition in Nepal 1998 25 157 172

[B17] Retherford RD Ramesh BM Fertility and contraceptive use in Tamil Nadu, Andhra Pradesh, and Uttar Pradesh NFHS Bulletin 1996 3 1 4 International Institute for Population Sciences 12291298

[B18] Beekle AT Mccabe C Awareness and determinants of family planning practice in Jimma, Ethiopia Int Nurs Rev 2006 53 269 276 10.1111/j.1466-7657.2006.00492.x 17083415

[B19] Bogale B Wondafrash M Tilahun T Girma E Married women's decision making power on modern contraceptive use in urban and rural southern Ethiopia BMC Public Health 2011 11 342 10.1186/1471-2458-11-342 21595897 PMC3114727

[B20] Dodoo FN Tempenis M Gender, power and reproduction differences in the relationship between fertility goals and contraceptive use in Kenya Royal Sociology 2002 67 1 46 70

[B21] Dodoo FN Men Matter: Additive and interactive gendered preferences and reproductive behavior in Kenya Demography 1998 35 2 229 242 10.2307/3004054 9622784

[B22] Do M Kurimoto N Women’s empowerment and choice of contraceptive methods in selected African countries Int Perspect Sex Reprod Health 2012 38 1 23 33 10.1363/3802312 22481146

[B23] Dyson T Moore M On kinship structure, female autonomy and demographic behavior in India Popul Dev Rev 1983 9 1 35 60 10.2307/1972894

[B24] Edwards SR The role of men in contraceptive decision-making: current knowledge and future implications Fam Plann Perspect 1994 26 2 77 82 10.2307/2136006 8033982

[B25] Ezeh AC The Influence of spouses over each other's contraceptive attitudes in Ghana Stud Fam Plann 1993 24 3 163 174 10.2307/2939231 8351697

[B26] Foreman M The Challenges of Integrating Family Planning and Maternal/Child Health Services, The Population Reference Bureau 2011

[B27] Jejeebhoy SJ Convergence and divergence in spouses' perspectives on women's autonomy in rural India Stud Fam Plann 2002 33 4 299 308 10.1111/j.1728-4465.2002.00299.x 12553187

[B28] Kamal N The influence of husbands on contraceptive use by Bangladeshi women Health Policy Plan 2000 15 1 43 51 10.1093/heapol/15.1.43 10731234

[B29] Speizer IS Nanda P Achyut P Pillai G Guilkey DK Family planning use among urban poor women from six cities of Uttar Pradesh, India J Urban Health 2012 89 4 639 658 10.1007/s11524-011-9667-1 22399250 PMC3535138

[B30] Balaiah D Ghule M Naik DD Parida RC Hazari KT Fertility attitudes and family planning practices of men in a rural community of Maharashtra J Fam Welfare 2001 47 1 56 67

[B31] Kanitkar T Kulkarni S Involvement of males in practice of contraception in Maharashtra Demography India 2002 31 1 1 16

[B32] Bruhin E Power, communication and condom use: patterns of HIV-relevant sexual risk management in heterosexual relationships AIDS Care 2003 15 389 401 10.1080/0954012031000105441 12828148

[B33] Dunkle KL Jewkes RK Brown HC Gray GE McIntryre JA Harlow SD Gender-based violence, relationship power and risk of HIV infection in women attending antenatal clinics in South Africa Lancet 2004 363 9419 1415 1421 10.1016/S0140-6736(04)16098-4 15121402

[B34] Pulerwitz J Amaro H De Jong W Gortmaker SL Rudd R Relationship power, condom use and HIV risk among women in the USA AIDS Care 2002 14 6 789 800 10.1080/0954012021000031868 12511212

[B35] UN Population Division World Urbanization Prospects: The 2007 Revision Population Database 2011 http://esa.un.org/unup/

[B36] Woolf SE Maisto SA Gender differences in condom use behavior? the role of power and partner-type Sex Roles 2008 58 689 701 10.1007/s11199-007-9381-3

[B37] Becker S Couples and reproductive health: a review of couple studies Stud Fam Plann 1996 27 6 291 306 10.2307/2138025 8986028

[B38] Becker S Fonseca BF Schenck YC Husbands' and wives' reports of women's decision-making power in Western Guatemala and their effects on preventive health behaviors Soc Sci Med 2006 62 9 2313 2326 10.1016/j.socscimed.2005.10.006 16307836

[B39] Ghuman Hedy L Herbert LS Measurement of women's autonomy according to women and their husbands: Results from five Asian countries Soc Sci Res 2006 35 1 1 28 10.1016/j.ssresearch.2004.06.001

[B40] Kulczycki A Husband-wife agreement, power relations and contraceptive use in Turkey Int Fam Plan Perspect 2008 34 3 127 137 10.1363/3412708 18957355

[B41] Miller K Zulu EM Watkins SC Husband-wife survey responses in Malawi Stud Fam Plann 2001 32 2 161 174 10.1111/j.1728-4465.2001.00161.x 11449864

[B42] Koffi AK Adjiwanou VD Becker S Olaolorun F Tsui AO Correlates of and couples’ concordance in reports of recent sexual behavior and contraceptive use Stud Fam Plann 2012 43 1 33 42 10.1111/j.1728-4465.2012.00300.x 23185870 PMC4140172

[B43] Stephenson R Bartel D Rubardt M Constructs of power and equity and their association with contraceptive use among men and women in rural Ethiopia and Kenya Glob Public Health 2012 7 6 618 634 10.1080/17441692.2012.672581 22568536

[B44] Singh A Reducing Gender Based Inequalities in Employment and Wages: Is India on Right Track? IGIDR 2012 Indira Gandhi Institute of Development Research

[B45] Teitelman AM Ratcliffe SJ Sullivan CM Sexual relationship power, intimate partner violence and condom use among minority urban girls J Interpers Violence 2008 23 12 1694 1712 10.1177/0886260508314331 18349344 PMC3677854

[B46] Barker G Contreras M Heilman B Singh A Verma R Nascimento M Evolving Men: Initial Results from the International Men and Gender Equality Survey (IMAGES) 2011 Washington, DC and Rio de Janeiro: International Center for Research on Women (ICRW) and Instituto Promundo

[B47] Verma RK Pulerwitz J Mahendra VS Khandekar S Singh AK Das SS Nura A Barkar G “Promoting Gender Equity as a Strategy to Reduce HIV Risk and Gender-Based Violence among Young Men in India” Horizons Final Report 2008 Washington, DC: Population Council

[B48] Puur A Livia SO Mariam IT Jurgen D Men's childbearing desires and views of the male role in europe at the dawn of the 21st century Demogr Res 2008 19 56 1883 1912 10.4054/DemRes.2009.21.3 PMC3882066 24403853

[B49] Saleem S Bobak M Women's autonomy, education and contraception use in Pakistan: a national study Reproductive Health 2005 2 1 1 8 16242030 10.1186/1742-4755-2-8 PMC1277848

[B50] Mason KO Smith HL Women’s Empowerment and Social Context: Results from Five Asian Countries 2003 World Bank, Washington, DC: Gender and Development Group

[B51] Mumtaz Z Salway S I never go anywhere': Extricating the links between women's mobility and uptake of reproductive health services in Pakistan Soc Sci Med 2005 60 8 1751 1765 10.1016/j.socscimed.2004.08.019 15686807

[B52] Stephenson R Hennink M Barriers to Family Planning Use Amongst the Urban Poor in Pakistan Opportunities and Choices 2004 University of Southampton, UK: Working Paper

[B53] Khan ME Anurag M Sudhakar M Exploring opportunities to project A ‘responsible man’ image: gatekeepers; views on young men’s sexual and reproductive health needs in Uttaranchal, India Int Q Community Health Educ 2008 28 1 13 31 18644762 10.2190/IQ.28.1.c

[B54] Jayraman A Mishra V Arnold F The effect of family size and composition on fertility desires, contraceptive adoption and method choice in South Asia Demographic and Health Research 2008 40 1 26 DHS Working Papers 10.1363/ifpp.35.029.09 19465346

[B55] Santhya K Changing Family Planning Scenario in India: An Overview of Recent Evidence 2003 17 New Delhi, India: Population Council, Regional Working Papers

[B56] Walque D Kline R Variations in condom use by type of partner in 13 Sub-Saharan African countries Stud Fam Plann 2011 42 1 1 10 10.1111/j.1728-4465.2011.00259.x 21500696

[B57] International Institute for Population Sciences (IIPS) & Macro International National Family Health Survey (NFHS-3), India, 2005–06: Uttar Pradesh 2008 Mumbai: IIPS

